# Glycolysis in tumor microenvironment as a target to improve cancer immunotherapy

**DOI:** 10.3389/fcell.2022.1013885

**Published:** 2022-09-19

**Authors:** Chu Xiao, He Tian, Yujia Zheng, Zhenlin Yang, Shuofeng Li, Tao Fan, Jiachen Xu, Guangyu Bai, Jingjing Liu, Ziqin Deng, Chunxiang Li, Jie He

**Affiliations:** ^1^ Department of Thoracic Surgery, National Cancer Center/National Clinical Research Center for Cancer/Cancer Hospital, Chinese Academy of Medical Sciences, Peking Union Medical College, Beijing, China; ^2^ Department of Colorectal Surgery, National Cancer Center/National Clinical Research Center for Cancer/Cancer Hospital, Chinese Academy of Medical Sciences, Peking Union Medical College, Beijing, China; ^3^ Department of Medical Oncology, National Cancer Center/National Clinical Research Center for Cancer/Cancer Hospital, Chinese Academy of Medical Sciences, Peking Union Medical College, Beijing, China

**Keywords:** glycolysis, cancer metabolism, TME, immunotherapy, immunity regulation

## Abstract

Cancer cells and immune cells all undergo remarkably metabolic reprogramming during the oncogenesis and tumor immunogenic killing processes. The increased dependency on glycolysis is the most typical trait, profoundly involved in the tumor immune microenvironment and cancer immunity regulation. However, how to best utilize glycolytic targets to boost anti-tumor immunity and improve immunotherapies are not fully illustrated. In this review, we describe the glycolytic remodeling of various immune cells within the tumor microenvironment (TME) and the deleterious effects of limited nutrients and acidification derived from enhanced tumor glycolysis on immunological anti-tumor capacity. Moreover, we elucidate the underlying regulatory mechanisms of glycolytic reprogramming, including the crosstalk between metabolic pathways and immune checkpoint signaling. Importantly, we summarize the potential glycolysis-related targets that are expected to improve immunotherapy benefits. Our understanding of metabolic effects on anti-tumor immunity will be instrumental for future therapeutic regimen development.

## Introduction

In the latest decade, immunotherapy has achieved great advances in cancer treatment in several malignancies (Pan et al., 2020). Immune checkpoint blockades (ICBs) and adoptive cell therapy (ACT)-based strategies have been approved as first-line therapies for various tumors (Al-Sawaf et al., 2019; Cohen et al., 2019; Reck et al., 2022). Despite the dramatic tumor regression in some patients accepting ICB therapy, many patients do not respond to these remedies initially (primary resistance), and a subset of patients responsive in the beginning develop resistance at a later time and undergo disease relapse, that is acquired resistance (Bagchi et al., 2021). Similarly, resistance and narrow application also limit ACT therapy effectiveness. Understanding the mechanisms of therapeutic resistance to immunotherapy has been listed as one of the top 10 challenges in cancer immunotherapy (Hegde and Chen, 2020). As the essential supporter of cancer cell viability and malignant processes, cancer metabolism, especially glucose metabolism, has been extensively studied for overcoming immunotherapy resistance (Bader et al., 2020).

Unlike normal cells, tumor cells prefer aerobic glycolysis to generate energy and meet other biological requirements of malignant phenotype (Chang et al., 2015). Researchers reported that the aberrant energy utilization mode of cancers could alter the TME, creating a tumor-favorable niche, and impairing effective cancer treatment (DePeaux and Delgoffe, 2021). In addition, metabolic rewiring was also observed in various tumor-infiltrating lymphocytes and myeloid cells, changing their cancer-killing or cancer-promoting effects (Leone and Powell, 2020; Aramini et al., 2022). Therefore, given the breakthrough research on the interplay between anti-tumor immunity and cancer metabolism reprogramming in the past decade, the combination strategy with immunotherapy and glycolysis-targeted therapy is emerging. Cascone et al. (2018) demonstrated that increased tumor glycolysis is associated with resistance to adoptive T cell therapy in melanoma and suggested the favorable benefits of dual targeting cancer immunity and metabolism. And glycolytic activity could upregulate immune checkpoint expression, thus promoting immunotherapy response (Jiang et al., 2019). These findings all validate that the manipulation of cancer cell and immune cell glycolytic metabolism, is actionable in optimizing cancer immunotherapy, but the detailed settings warrant further exploration. It is should be noted that different cancer types and even stratified cancer cell clusters in the same tumor tissue have heterogeneous metabolic activity and dependency (Li et al., 2022b), which may confer distinct vulnerability and responses of cancers to immunotherapy and glycolytic-targeted therapy (Wang et al., 2022a). Here, we elucidate the roles glycolytic reprogramming within TME plays in cancer immunity, and we highlight the potentiality of targeting glycolysis in enhancing immunotherapy.

## The glucose metabolism features in tumor microenvironment

### Glucose metabolism in cancer cells

Normal cells utilize mitochondrial respiration, termed oxidative phosphorylation (OXPHOS), to sustain bioenergy synthesis and viability. Conversely, in response to hypoxia and oncogenic signals, like MYC and PI3K, cancer cells predominantly utilize glycolysis to support rapid growth and genome replication, even under abundant oxygen (Chang et al., 2015). This metabolic reprogramming is called the “Warburg effect” or aerobic glycolysis, a prominent feature of energy metabolism in cancer.

The glycolytic process is composed of glucose transporter 1 (GLUT1)-mediated glucose uptake and the conversion from glucose into pyruvate through ten consecutive enzymatic reactions in an oxygen-independent manner. Newly generated pyruvate is either catalyzed in acetyl-CoA to fuel the tricarboxylic acid (TCA) cycle or is reduced toward lactate by lactate dehydrogenase (LDH) to achieve the regeneration of NAD^+^ for continuous glycolysis and NAD^+^/NADH redox balance (Chang and Pearce, 2016). Redundant lactate is released from cells by monocarboxylate transporters (MCTs). In addition to energy production, glycolysis also provides biosynthesis precursors to cross-link other metabolic pathways, such as 3-phosphoglycerate for one-carbon pathway, thus playing a critical role in nucleotide and amino acid synthesis (Leone and Powell, 2020).

### Glucose metabolism in immune cells

**FIGURE 1:**
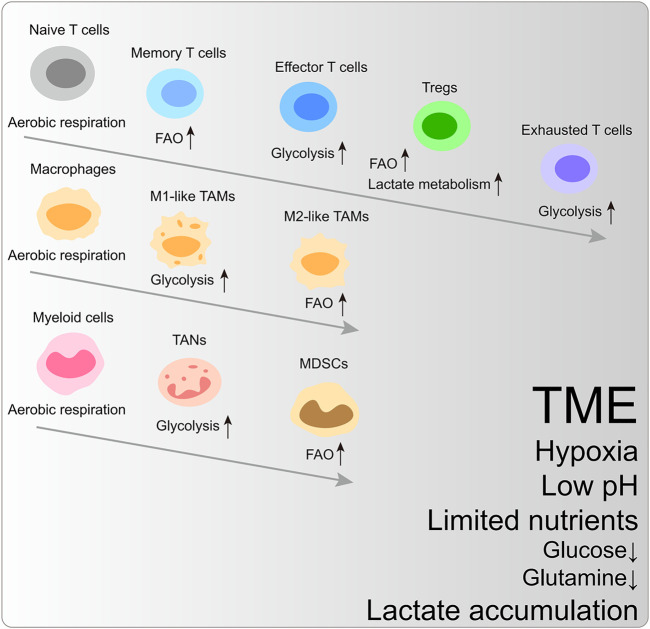
The metabolic reprogramming of immune cells within the characteristic tumor microenvironment. FAO, fatty acid oxidation; Tregs, regulatory T cells; TAM, tumor-associated macrophages; TAN, tumor-associated neutrophils; MDSCs, myeloid-derived suppressor cells.

Glycolytic preference is a shared mechanism to satisfy biosynthesis and energy requirements in rapidly proliferating cells. In the TME, cancer cells, effector T cells, and M1-like macrophages are prone to upregulate glycolysis and glutaminolysis fluxes. In contrast, memory T cells, regulatory T cells (Tregs), and M2-like macrophages mainly rely on fatty acid oxidation (FAO) (Figure 1) (Andrejeva and Rathmell, 2017).

Naïve T cells utilize TCA-coupled OXPHOS to maintain hypometabolic status. Upon MHC-peptide stimulation, the T cell receptor (TCR) coupled with CD28 activates PI3K-AKT-mTORC1 and MYC signaling pathway to trigger metabolic reprogramming (Wang et al., 2011). To enhance anabolic metabolism for cancer-killing function and clone expansion, effector T cells are metabolically activated and upregulate aerobic glycolysis. Simultaneously, the intermediates from glycolysis are essential for T cell effector activation and cytokine generation. For example, glycolytic metabolite phosphoenolpyruvate (PEP) blocks Sarco/endoplasmic reticulum Ca^2+^-ATPase (SERCA)-mediated ER calcium uptake to retain cytosol Ca^2+^ and nuclear factor of activated T-cells (NFAT) signaling, which is indispensable for TCR signaling transduction (Ho et al., 2015). Phosphoenolpyruvate carboxykinase 1 (PCK1) can catalyze oxaloacetate (OAA) into PEP, and PCK1 overexpression boosts the adoptive transferred CD4^+^ and CD8^+^T cell cancer-killing functions (Ho et al., 2015).

Exhausted CD8^+^T cells prefer glycolysis to OXPHOS, and this metabolic adaptation to TME further dampens their cancer-inhibiting function (Scharping et al., 2021). Memory T cells remain in metabolically quiescent states and depend on FAO to fuel OXPHOS (Pearce et al., 2013). However, glycolysis is unnecessary for T cell persistence fed by general glucose metabolism (Chang et al., 2013).

The functions of CD4^+^T cells are also intertwined with specific metabolic reprogramming. Glycolysis promotes the production of IL-2, TNFα, and IFNγ in CD4^+^T cells, while whose repression can lead to CD4^+^T cell hyporesponsiveness and exhaustion, along with increased expression of PD-1 and LAG-3 (Jancewicz et al., 2021; Martins et al., 2021). Inflammatory CD4^+^T cells (Th1 and Th17 cells) have high glucose activity. The pyruvate dehydrogenase (PDH) inhibitor, pyruvate dehydrogenase kinase isozyme 1 (PDHK1), is exclusively expressed in Th17 cells, whose ablation changes the immune cell composition, including selective reduction of Th17 cells (Gerriets et al., 2015). Instead, Tregs ramp up OXPHOS and FAO to maintain bioenergy synthesis (Gerriets et al., 2015). The Tregs-specific expression of transcription factor FOXP3 restrains MYC signaling and antagonizes PI3K-AKT-mTORC1 axis-mediated glycolytic activation to increase oxidation and catabolic metabolism, endowing the survival advantage for Tregs in TME (Gerriets et al., 2016; Angelin et al., 2017). Moreover, Tregs can utilize lactate-metabolic pathways to sustain proliferation and suppressive identity in the low-glucose condition. High-glucose *ex vivo* culture condition even dampens their stability (Watson et al., 2021). These data indicate the difference in functionally differentiated T cells’ metabolic signature.

M1-like macrophages mainly rely on glycolysis to sustain inflammatory phenotype (Kelly and O'Neill, 2015). Instead, M2-like macrophages exhibit an immunosuppressive phenotype, depending on the TCA cycle and FAO (Netea-Maier et al., 2018). A study found that the acidic living environment of highly glycolytic melanoma further induced tumor-associated macrophages (TAMs) toward a cancer-promoting phenotype (Bohn et al., 2018).

The intrinsic heterogeneity of tumor-associated neutrophils (TANs) and cancer contexts determine these myeloid cells’ pro- or anti-tumor effects. In pancreatic ductal adenocarcinoma (PDAC), the multi-omics approach exhibits that TANs undergoing glycolytic switch mediated by LDHA upregulation showed tumor-promoting phenotype (Wang et al., 2022b). Triple-negative breast cancer (TNBC) cells with accelerated glycolysis highly express granulocyte colony-stimulating factor (G-CSF) and granulocyte-macrophage colony-stimulating factor (GM-CSF) under the regulation of liver-enriched activator protein and AMP-activated protein kinase (AMPK)-serine/threonine-protein kinase (ULK1) pathway, supporting myeloid-derived suppressor cells (MDSCs) development and facilitating CD8^+^T cell inhibition and cancer progression (Li et al., 2018). Moreover, increased lactate generation enhances the tumor-promoting capacity of MDSCs through the G protein-coupled receptor 81 (GPR81)/mTOR/HIF-1α/STAT3 pathway in PDAC (Yang et al., 2020).

Of note, TME is characterized by loss of essential nutrients, inadequate vascularization, lactate accumulation, and hypoxia, and all these detrimental conditions contribute to cancer-killing T cell disability to a large extent. Here we focus on the direct results of the increased glycolytic flux, including limited glucose and acidic TME.

### Glucose deficiency

Cancer cells with activated glycolysis outcompete immune cells for glucose utilization, and glucose deficiency impairs the persistence and function of effector T cells. For example, the expression of glycolysis-related genes, such as ALDOA, ALDOC, ENO2, GAPDH, GPI, and PFKM, negatively correlates with T cell infiltration in TCGA cohorts of melanoma and NSCLC patients (Cascone et al., 2018). The restricted glucose supplement in TME directly damages the glycolytic capacity of effector T cells (Ho et al., 2015), and T cells with low glycolytic potency exhibit exhausted states and reduced anti-apoptosis gene and effector gene expression (Song et al., 2022a). In the mice sarcoma model, glucose deprivation could inhibit the mTOR activity and IFN-γ production of tumor-infiltrating lymphocytes (TILs) (Chang et al., 2015). In addition, glucose absence also disturbs mitochondrial functions. Mitochondrial abnormalities can promote the permanent terminal exhaustion of CD8^+^T cells (Yu et al., 2020).

### Tumor microenvironment acidification

Beyond the fierce competition of glucose between cancer cells and TILs, the lactate generated from intensive glycolysis also contributes to immunodepressive TME. Lactate metabolism is involved in oncogenesis (Kooshki et al., 2022), especially the serum lactate concentration is positively associated with cancer burden (Fischer et al., 2007), as well as LDH level is a biomarker for poor prognosis and immunotherapy efficacy (Robert et al., 2022). Low pH (6.5–6.9) is adverse to anti-tumor immunity, where effector CD8^+^T cells exhibit reduced IL-2Rα (CD25) and TCR expression, along with STAT5 and extracellular signal-regulated kinase (ERK) inactivation (Calcinotto et al., 2012). Lactate also disturbs the TCR signaling by blocking the phosphorylation of JNK, c-Jun, and p38 (Mendler et al., 2012). Exposed to a large amount of lactate, Tregs prefer OXPHOS to regenerate NAD^+^ through abundant lactate converted into pyruvate. In contrast, effector T cells are hard to maintain the balance of NAD^+^ to NADH in this way (Angelin et al., 2017). In addition, lactate is a mediator to promote the expression of proinflammatory cytokines in tumor-infiltrating immune cells, including IL-23 and IL-17, thereby motivating tumorigenesis and impairing anti-tumor activity (Shime et al., 2008). Acidification also impairs the antigen presentation processes, including antigen-MHC-Ⅰ-complexes stability and cross-presentation of DCs, and is correlated with poor clinical benefits of DC vaccines (Caronni et al., 2018; Burgdorf et al., 2020). Previous studies indicated that acidification neutralization by bicarbonate could enhance T cell functional reversion and infiltration (Mendler et al., 2012; Pilon-Thomas et al., 2016). Notably, it has been reported that the type-Ⅰ IFN, which is downstream of TLR3 and STING, can be inhibited in the lactate-abundant TME. It is a novel viewpoint about the role of glycolysis in innate anti-tumor immunity (Caronni et al., 2018).

### Hypoxia

Hypoxia is an essential feature of TME, resulting from poor vascularization and high metabolism of cancer cells. Enhanced glycolysis is partially attributed to oxygen-limited in the TME. HIFs promote glycolytic gene transcription to maintain the anaerobic metabolism of cancers and immune cells (Smith et al., 2016; Cho et al., 2019; Bader et al., 2020). As an environmental stimulator, hypoxia has a conflicting effect on anti-tumor immunity. Effector T cells would undergo epigenetic reprogramming upon hypoxia exposure, and the altered epigenetic regulation reduces their transcription and function (Ford et al., 2022; Ma et al., 2022). In contrast, another study demonstrated that hypoxia-inducible factor-1α (HIF-1α) deletion led to the reduction in T cell infiltration and tumor killing during hypoxia conditions (Palazon et al., 2017). While under oxygen limitation, HIF-1α can be induced in Tregs, and bind to the promoter region of the FOXP3 to promote transcription (Clambey et al., 2012). HIF-1α also favors an immunosuppressive TME by reinforcing other cancer-promoting immune cell functions, including MDSCs and M2-like macrophages (Deng et al., 2019; Zhang et al., 2022). Overall, hypoxia promotes glycolysis flux in cancer cells and makes negative effects on cancer immunity to a greater extent than positive effects.

## The regulatory signaling pathways of glycolysis

Targeting critical glycolytic regulation signaling pathways which modulate cancer cell and immune cell glycolysis reprogramming is a promising approach for enhancing the anti-tumor capability (Bader et al., 2020), and several related drugs like metformin and phenformin have been extensively tested in clinical trials (García Rubiño et al., 2019; Chow et al., 2022)

### LKB1-AMPK signaling pathway

The liver kinase B1 (LKB1)-AMPK pathway regulates cell metabolism according to different energy statuses (Blagih et al., 2012). Upon stimulation, the LKB1-dependent kinases can regulate downstream metabolic pathways by targeting multiple effectors, including AMPK (Shackelford and Shaw, 2009). AMPK has three subunits, α, β, and γ. The β subunit binds with glycogen particles, and the γ subunit has two mutually antagonistic nucleotide binding sites for AMP and ATP. These structures allow AMPK to sense bioenergy fluctuation and mediate energy metabolism switch from anabolism toward catabolism to maintain energy homeostasis (Dërmaku-Sopjani and Sopjani, 2019).

**FIGURE 2:**
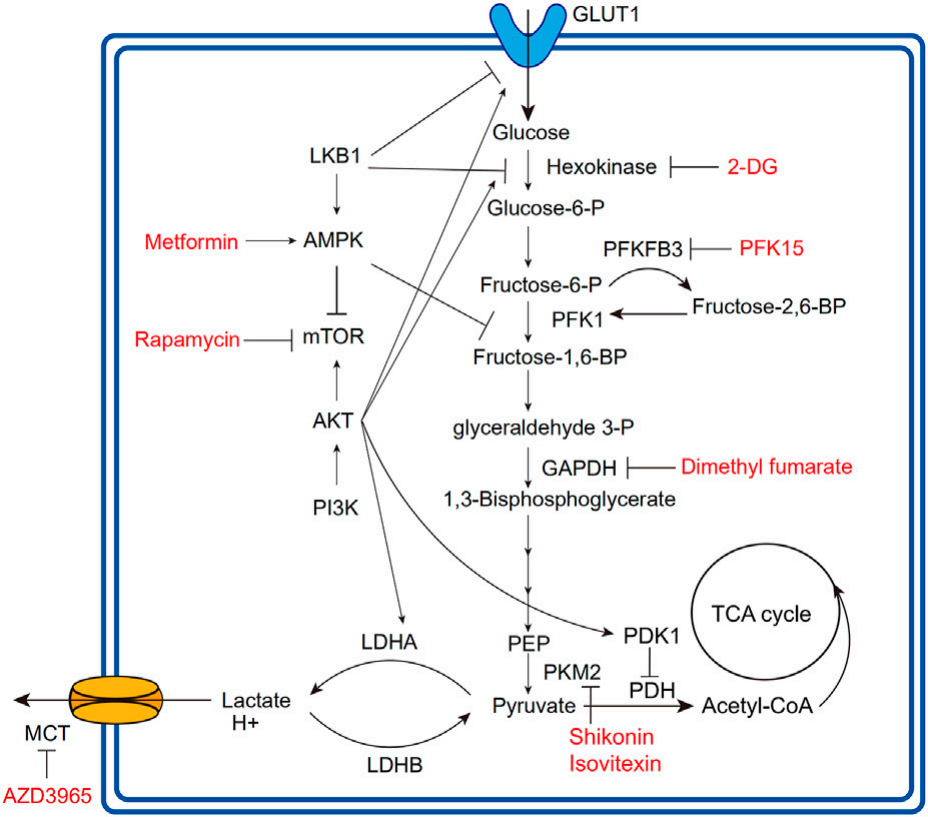
Overview of glycolytic process and regulatory relationships. The fonts marked red are agents developing in clinical or preclinical trials for targeting corresponding regulatory molecules. The lines with arrows mean upregulation or activation; the lines without arrows mean downregulation or repression. PFK1, phosphofructokinase1; PEP, phosphoenolpyruvate; PDK1, pyruvate dehydrogenase kinase1; PDH, pyruvate dehydrogenase; PKM2, pyruvate kinase isoform M2.

Loss of LKB1 increases glycolytic transcription and flux in T cells by mechanisms including upregulated expression of GLUT1 and hexokinase2 (HK2) (Figure 2) (MacIver et al., 2011). Intriguingly, under glycolysis inhibition by 2-Deoxy-D-glucose (2-DG) or IL-2-deficiency, LKB1 ablation significantly induces T cell death, indicating the impaired stress response of T cells (MacIver et al., 2011). In parallel with LKB1, AMPK maintains T cell mitochondrial bioenergetics and ATP production following pathogen infections, meanwhile regulating CD8^+^T cell primary response and Th1 and Th17 differentiation (Blagih et al., 2015). Antagonizing the effects of the PI3K-AKT-mTOR axis (Shackelford and Shaw, 2009), AMPK is activated in low-glucose TME to inhibit glycolysis-related gene expression, such as GLUT1, whereby downregulating glycolytic flux and promoting cellular metabolism toward catabolism and OXPHOS, in favor of immunosuppressive immune cells including Tregs, M2-like macrophages, and MDSCs (Michalek et al., 2011; Bader et al., 2020). Moreover, AMPK also phosphorylates phosphofructi-2 kinase (PFK2) to modulate glycolytic activity (Almeida et al., 2004).

### PI3K-AKT-mTOR signaling pathway

The PI3K-AKT-mTOR axis is extensively involved in cell proliferation, survival, cell cycle, and glucose metabolism (Thorpe et al., 2015). As a major glycolysis-promoting signaling pathway, the PI3K-AKT-mTOR axis deregulation can promote HIF-1α activation, GLUT1 expression, and several metabolic enzyme activations (Figure 2) (Denko, 2008; Icard et al., 2022).

In TCR-stimulating T cells, PI3K signaling increases AKT phosphorylation and glycolytic flux via LDHA (Xu et al., 2021a). AKT is the major regulator for glycolysis in cancer and immune cells. AKT activation upregulates the expression of GLUT1 and LDHA (Jacobs et al., 2008), promotes the phosphorylation of HK2 and PFK2 to activate HK1 (Miyamoto et al., 2008), and inhibits PDH by activating pyruvate dehydrogenase kinase-1 (PDK1) to promote the glycolytic process (Chae et al., 2016). HIF-1α is the major transcription factor regulated by mTOR, and many glycolytic enzymes, as well as the GLUT family, are the downstream target of HIF-1α (Denko, 2008). Of interest, mTOR kinase is found to determine the fate of effector and memory CD8^+^T cells. Blocking mTOR activity by rapamycin can promote memory cell precursors (Araki et al., 2009), especially the newly differentiated memory CD8^+^T cells equipped with more superior anti-tumor activity than IL-2-inducing effector T cells (Rao et al., 2010). mTOR activation also promotes T cell effector functions. For example, in LKB1/AMPK deficient T cells, mTOR upregulation propels IFNγ production (MacIver et al., 2011). Dotsu et al. introduced a small molecule PQDN that can activate T cells following TCR stimulation and demonstrated that PQDN could augment CD8^+^T cell function through AKT-mTOR activation (Dotsu et al., 2022).

## Targeting glycolysis in combination with immunotherapy

### Adoptive cell therapy

ACT boosts anti-tumor immunity by directly providing therapeutic modified immune cells. T cells are representative cell types for the ACT, including *ex vivo* expanded TILs, and engineered T cells expressing antigen-specific TCRs or chimeric antigen receptors (CARs) (Madden and Rathmell, 2021). ACT has achieved remarkable success in several cancer types, like CD19-targeted CAR-T cells in B cell-derived hematological malignancies. People can modulate the functional and differential states of T cells prior to cell infusion to make them more lethal to cancer.

Metabolic modulation is critical for promoting tumor cytotoxicity and the effectiveness of ACT. Therefore, enhancing the metabolic adaptation of therapeutically used T cells in the nutritionally competitive TME is promising to improve ACT efficacy (Sudarsanam et al., 2022). Particularly, glycolysis has unfavorable effects on these ACT-used T cells during the expansion phase and intratumoral function. For example, less differentiated CAR-T cells have superior persistence and anti-tumor ability, whereas high glycolytic flux is against developing long-lasting memory phenotypes for T cells (Madden and Rathmell, 2021). Adding glutamine antagonist 6-Diazo-5-oxo-l-norleucine (DON) into the culture medium can enhance CAR-T cell FAO and reduce glycolysis. In this way, CAR-T cells remain in more undifferentiated states (Shen et al., 2022). Interestingly, in the culture medium for CAR-T cell *ex vivo* expansion, IL-2, IL-7, and IL-15 are commonly added cytokines (Sudarsanam et al., 2022). IL-2 can propel terminal differentiation and enhance aerobic glycolysis of CD8^+^T cells, consequently leading to poor efficiency after adoptive transfer (Hermans et al., 2020).On the contrary, IL-21 upregulates the OXPHOS utilization and stem-like phenotype of CAR-T cells (Hermans et al., 2020). IL-7-secreting CD4^+^CAR-T cells have lower metabolic activity in the resting stage and respond to neoplastic stimulation with faster metabolic activation (Li et al., 202a). The influence of culture medium supplements on CAR-T cell metabolism is worthy of more attention to optimize therapeutic efficiency.

Moreover, studies focusing on the outcome of combining glycolysis inhibition with ACT have further proved the detrimental effects of the glycolytic pathway on ACT cells. HK2 inhibitor 2-DG upregulates AMPK phosphorylation which negatively regulates mTOR activity to shut down glycolysis in adoptive transfer CD8^+^T cells (Sukumar et al., 2013). These CD8^+^T cells treated with 2-DG express more memory phenotype-related markers, and glycolysis inhibition can promote the proportion and quality of memory CD8^+^T cells even during T cell priming (Sukumar et al., 2013). In agreement with the PI3K-AKT-mTOR pathway is pivotal in activating aerobic glycolysis, CD19-CAR-T cells have remarkable central-memory phenotypes and more robust tumor elimination efficacy when treated with AKT inhibitors (Klebanoff et al., 2017). Oppositely, mitochondrial oxidation is necessary for long-term survival and the maintenance of the memory-like phenotype of CAR-T cells. Therefore, rewiring mitochondria, such as enhancing mitochondrial lipid metabolism and oxygen consumption, is a feasible strategy to improve CAR-T cell superiority (Madden and Rathmell, 2021). Beyond the direct glycolysis inhibition, short-term culture, less glucose, and the inosine inhibitors addition are also helpful for CAR-T cells to develop a desirable phenotype (Sudarsanam et al., 2022). Transient glucose restriction (TGR) can enhance the anabolic program and tumor clearance efficiency of effector CD8^+^T cells after glucose re-exposure (Klein Geltink et al., 2020). Intracellular lactate accumulation also impedes T cell proliferation and NAD^+^ regeneration (Quinn et al., 2020). Thus LDH inhibition is proposed as a feasible strategy to optimize CAR-T cell therapy (Hermans et al., 2020; Mane et al., 2020).

### Immune checkpoint blockades therapy

Chronic antigen stress and the immunosuppressive molecules within the TME can focus cytotoxic T cells to an exhausted status, characterized by immune checkpoint expression and impaired cytotoxic effects. Checkpoint molecules, such as CD28, CD40L, and cytotoxic T lymphocyte-associated protein 4 (CTLA-4), cooperate with TCR signaling to initiate activation, persistence, and exhaustion of TILs. Functional alternations during the exhaustion process are concurrent with metabolic adaptations, which further support the immunosuppressive functions of checkpoints. Therefore, targeting critical glycolytic regulators or metabolites that contribute to immune checkpoints-mediated T cell inhibition is a promising therapeutic strategy in combination with ICBs.

Programmed cell death protein 1 (PD-1) and CTLA-4 are two well-known immune checkpoints. CTLA-4 only expresses on T cells, plays an immunosuppressive role in the initial phase when naïve T cells are activated by antigen-presenting cells (APCs) in lymphoid tissues, and impedes primary T cell activation-induced glycolysis (Rudd et al., 2009). PD-1 expresses following T cell activation in peripheral tissues, as a marker of activated T cells and early exhausted T cells (Morad et al., 2021). PD-1 and CTLA-4 can impair the metabolic reprogramming induced by CD28 co-stimulation. Mechanistically, CD28 signaling senses ATP/ADP levels in T cells to control glucose uptake, and then activates the PI3K-AKT signaling pathway that increases glycolytic flux (Frauwirth et al., 2002). CTLA-4 competes with CD28 for binding to CD80/CD86 and inhibits the downstream signaling of CD28. The YVKM motif of CTLA-4 is bound by PI3K and phosphatases SHP-2 and PP2A with negative signaling to inhibit T cell activation (Parry et al., 2005; Rudd et al., 2009). CTLA-4 blockades can enhance the metabolic fitness of CD8^+^T cells and destabilize Tregs in glycolysis-defective tumors, indicating that the combination of CTLA-4 blockers and glycolysis inhibitors is a promising strategy in cancer (Zappasodi et al., 2021). Additionally, when effector T cells are activated, PD-1 signaling inhibits glycolysis and induces OXPHOS dysregulation by interrupting AKT phosphorylation and recruiting SHP1/2 phosphatase to inhibit PI3K activation before T cells show significant incapacitation (Parry et al., 2005; Bengsch et al., 2016; Ogando et al., 2019). PD-1 also promotes FAO by upregulating CPT1A expression in T cells (Patsoukis et al., 2015). Tkachev et al. found that during allogeneic bone marrow transplantation, PD-1 could increase ROS by FAO to destroy alloreactive T cell survival (Tkachev et al., 2015). Of note, a recent study found that Tregs enhanced lactate absorption and then upregulated the PD-1 expression competing out effector T cells in glucose-limited TME, and in this setting anti-PD-1 therapy promoted Treg function, suggesting a novel immunotherapy resistance mechanism (Kumagai et al., 2022).

In addition to PD-1 and CLTA-4, there are various targetable coinhibitory molecules or positive immune regulators participating in cancer and immune cells’ metabolic rewiring (Morad et al., 2021). High T-cell immunoglobulin mucin receptor 3 (TIM-3) expression in a large subset of tumor-infiltrating Tregs can enhance Tregs’ repressive functions by upregulating glycolysis (Banerjee et al., 2021). TIM-3 is also highly expressed in human myeloid leukemia cells, mediating PI3K-mTOR and hypoxic pathway activation to enhance glycolysis (Prokhorov et al., 2015), In contrast, another study found that glycolytic flux negatively correlates with TIM-3 expression in Jurkat T cells (Lee et al., 2020), so the association between TIM-3 and glycolysis warrants further illustration. These findings indicate that TIM-3 has effects on the metabolism pathway in both lymphoid and myeloid lineages. The co-stimulator OX40 is also positively correlated with glycolysis and fatty acid metabolism activity in Tregs (Pacella et al., 2018). V-type immunoglobulin domain-containing suppressor of T-cell activation (VISTA) is another inhibitory checkpoint molecule. And the co-inhibitory receptor P-selectin glycoprotein ligand-1 (PSGL-1) can bind to the extracellular domain of VISTA and repress T cell function in the acidic TME (Johnston et al., 2019). 4-1BB (CD137) is a stimulator for CD8^+^T cell proliferation, and its agonist significantly activates the glycolysis, mitochondrial functions, and fatty acid metabolism of T cells by increasing GLUT1 expression and activating the AMPK-acetyl-CoA carboxylase (ACC) pathway (Choi et al., 2017). Based on the positive association between 4-1BB signaling and the TILs biogenesis, 4-1BB agonist in combination with adoptive cell therapy and PD-1 blockade can further augment cancer elimination based on immunotherapy potency (Menk et al., 2018). Likewise, the immune co-stimulator (ICOS) can also activate mTORC1 and mTORC2 to drive glycolysis and lipogenesis of follicular helper T cells and enhances their responses (Zeng et al., 2016).

Collectively, metabolic dysfunction is an essential mechanism through which immune checkpoint molecules constrain anti-tumor immunity. The combinatorial regimen of ICBs and metabolic regulators is a promising therapeutic strategy. Glycolysis-targeted agents which show favorable efficacy in combination with immunotherapies are listed in Table 1.

**TABLE 1:** The combination regimens for glycolysis-targeted agents and immunotherapies in preclinical studies.

Target	Human cancers/murine cancer models	Agents	Mechanisms	Immunotherapy	References
Hexokinase	Breast cancer, ovarian cancer	2-DG, Lonidamine	Inhibiting Treg metabolism reprogramming in TME; derepressing T cell proliferation; Upregulating PD-L1 expression in cancer cells	Anti-CTLA-4/PD-1 therapy	Xu et al., 2021a; Guo et al., 2022; Luo et al., 2022
GAPDH	Breast cancer, colon adenocarcinoma	Dimethyl fumarate	Promoting oxidative pentose phosphate pathway in cancer cells and inhibiting cancer cells’ competition for glucose	ICB/IL-2 therapy	Lei et al. (2022)
Acidified TME	Melanoma, pancreatic carcinoma	PPI(Esomeprazole), Bicarbonate	Buffering the acidification TME	ACT/anti-CTLA-4/anti-PD-1 therapy	Calcinotto et al., 2012; Pilon-Thomas et al. (2016)
PFKFB	Hepatocellular carcinoma, myeloma, breast cancer	PFK15	Disturbing the metabolic support of cancer-associated fibroblasts for cancer cells; Inhibiting PD-L1 expression in cancer cells and macrophages	Anti-PD-1 therapy	Chen et al., 2019; Liu et al., 2022; Zang et al., 2022
PKM2	Pancreatic carcinoma, lung cancer, melanoma	Shikonin, isovitexin	Reducing PD-L1 expression	Anti-PD-1/PD-L1 therapy	Zhao et al., 2018; Chen et al., 2021a
NAD^+^	Melanoma, colon tumor	NAD precursor (nicotinamide riboside)	NAD^+^ reduces mitochondria abnormality in TILs	Anti-PD-1 therapy	Yu et al. (2020)
LDH	Melanoma, non-small cell lung cancer	Oxamate	Impeding IL-2-induced CD8^+^T cell terminal differentiation; Increasing T cell tumor infiltration	ACT/anti-PD-1 therapy	Mane et al., 2020; Qiao et al. (2021)
MCT1	Melanoma, head and neck squamous cell carcinoma, colon carcinoma	AZD3965	Repressing lactate uptake and PD-1 expression in Tregs; Reducing TME acidification	Anti-PD-1 therapy	Huang et al., 2021; Watson et al. (2021)
MCT4	Hepatocellular carcinoma	VB124	Promoting effector T cell infiltration; reducing acidification	Anti-PD-1 therapy	Fang et al. (2022)

## Available glycolysis-targeted therapies

### mTOR

As an oncogenic signaling pathway, direct inhibition of the mTOR axis regulates the metabolic characteristics of tumor-infiltrating immune cells and blocks the malignant phenotype of cells. Based on their inhibitory effects on cancer metabolism and growth, rapamycin analogs have been approved for cancer treatment (Holloway and Marignani, 2021). However, these agents are likely to diminish anti-tumor immunity (Bader et al., 2020). The glycolytic activity affected by rapamycin is the pivotal determinant for lineage differentiation (Shi et al., 2011). Rapamycin suppresses the Th17 differentiation and promotes Tregs differentiation under the induction of TGFβ (Kopf et al., 2007). Studies found that enforced AKT-mTORC1 signaling can restore T cell function and reduce the expression of immune checkpoint molecules such as PD-1 and TIM-3 to retard T cell exhaustion (Siska et al., 2016). And mTORC1 overactivation impairs the immunosuppressive capacities of Tregs, whereas a low level of mTORC1 can enhance Tregs activity (Chapman et al., 2018). Therefore, identifying the most optimized inhibitory and activated level of the PI3K-AKT-mTOR axis is significant to the efficiency of mTOR-related therapy.

### AMP-activated protein kinase

As a classical activator of AMPK, metformin has exhibited satisfactory treatment effects in various cancer types (Chow et al., 2022). Metformin regulates metabolic signature by interacting with the PI3K-AKT-mTOR axis and HIF-1α (Nozhat et al., 2018; Shao et al., 2020), both of which are the main regulators of Warburg effects. A bulk of studies have validated that metformin also promotes the cancer-killing functions of CD8^+^T cells in metabolic regulation-dependent manners (Pearce et al., 2009; Chen et al., 2021b; Chao et al., 2022). Metformin treatment downregulates immune checkpoint expression and glycolytic cancer flux in a HIF-1α inhibition-dependent manner, thereby improving ICB therapy (Chung et al., 2021; Song et al., 2022b). Furthermore, the metabolic rewiring effects-mediated by metformin can affect the metabolic interaction between cancer cells and non-malignant cells within TME. For example, lactate and ketone bodies produced by cancer-associated fibroblasts (CAFs) produce can be utilized by cancer cells in the TME. Metformin disturbs the cross-feeding of CAFs and cancer cells to repress the additional nutrient support for cancers (Mostafavi et al., 2022).

### Hexokinase

2-DG is an effective HK inhibitor to repress the Warburg effect phenotype of cancers (Pouysségur et al., 2022). Luo et al. (2022) designed a novel multi-targeted nano drug termed D/B/CQ@ZIF-8@CS enveloping 2-DG, BAY-876 (GLUT1 inhibitor), and chloroquine (CQ), which sufficiently inhibits the aerobic glycolysis procedure of cancer cells. Significantly, the drug synthetically improves the anti-CTLA-4 immunotherapy efficacy by reducing Tregs metabolic fitness in the relieved TME, which is used to be glucose-deficient and lactate-enriched (Luo et al., 2022). In ovarian cancer, Tregs pretreated with 2-DG also alleviate the inhibition of effector T cell proliferation (Xu et al., 2021b). In addition, HK recently has been found to upregulate PD-L1 expression in cancer cells in an NF-κB-dependent pathway, and HK inhibitor Lonidamine has shown favorable cancer elimination in combination with anti-PD-1 therapy in mice model (Guo et al., 2022). However, the severe systemic toxicity induced by HK inhibitors in clinical studies warrants further investigation (Pouysségur et al., 2022).

### Glucose-6-phosphate isomerase

Glucose-6-phosphate isomerase (GPI) catalyzes glucose-6-phosphate (G6P) toward fructose-6-phosphate (F6P), and its overexpression is documented in various cancer types, such as lung adenocarcinoma (Han et al., 2021) and gastric cancer (Ma et al., 2018), associated with highly activated Warburg effect phenotype of cancer cells and poor prognosis. GPI knockout experiments demonstrate that cancer cells will motivate OXPHOS following GPI KO-induced glycolytic inhibition to sustain survival. Still, GPI silencing combined with mTORC1 and OXPHOS inhibition significantly represses tumor growth (Pouysségur et al., 2022). GPI inhibition is found to determine autoimmunological-related and homeostatic-related Th17 immune cell persistence in a context-dependent manner (Wu et al., 2020), but the evidence explaining the role of GPI silencing in anti-tumor immunity is absent. Whether GPI-targeted metabolic reprogramming can enhance immunotherapy is explored further.

### Glyceraldehyde-3-phosphate dehydrogenase

Glyceraldehyde-3-phosphate dehydrogenase (GAPDH) reduces glyceraldehyde-3-phosphate toward 1,3-bisphosphoglycerate within the glycolytic pathway. GAPDH inhibitor, Dimethyl fumarate (DMF), is reported to facilitate the oxidative pentose phosphate pathway (PPP) while inhibiting glycolysis and OXPHOS in tumor cells. The reduced glucose competition between cancer cells and T cells mediated by DMF improves the efficacy of ICB and IL-2 therapy in BRCA and COAD patients (Lei et al., 2022). Intriguingly, low-dose osimertinib can inhibit GAPDH activity and tumor endothelial glycolysis, thereby promoting adequate vascularization and immune cell infiltration to improve the tumor repression efficacy of PD-1 blockade (Shan et al., 2022).

### Fructose-2,6-bisphosphatase

Fructose-2,6-bisphosphatase (PFKFB) catalyzes the synthesis and degradation of fructose 2-6 biphosphate (F-2,6-BP), the positive allosteric effector of glycolytic rate-limiting enzyme PFK. Zheng et al. (2022) found that fructose-2,6-bisphosphatase 3 (PFKFB3) is upregulated in various cancer types, and its inhibition represses the glycolysis of cancer cells and concomitantly upregulates PD-L1 expression. Furthermore, glucose deficiency can reversibly upregulate PD-L1 expression through EGFR/ERK/c-Jun pathway in cancer cells and then increased PD-L1 upregulates PFKFB3 to promote glycolysis (Chen et al., 2019; Liu et al., 2022). The finding uncovers a new therapeutic resistance mechanism: checkpoint molecule and glycolytic metabolism construct an immunosuppressive positive feedback loop (Yu et al., 2021). Zang et al. (2022) designed a dual-target drug of paclitaxel and PFKFB3 inhibitor PFK15 to block the CAF-mediated metabolic support for cancers. The drug synthetically inhibits cancer growth and concomitantly reduces lactate concentration in the TME. Moreover, PFK15 cripples diabetogenic CD4^+^T cell effects by leading to metabolic expression and upregulating PD-1 and LAG-3 expression in the context of Type 1 Diabetes, suggesting the considerable role of PFK15 plays in T cell immunogenetic regulation (Martins et al., 2021). Combined with anti-PD-1 treatment, the efficacy of PFKFB3 inhibitors can be enhanced in cancer therapy (Zheng et al., 2022).

### Pyruvate kinase isoform M2

Pyruvate kinase isoform M2 (PKM2) is the critical enzyme for the final rate-limiting step of glycolysis, converting phosphoenolpyruvate toward pyruvate. Palsson-McDermott found that PKM2 accelerates tumor progression by promoting PD-L1 expression in macrophages, DCs, and tumor cells (Palsson-McDermott et al., 2017). In PDAC, the high expression of PKM2 is a poor prognostic factor. PKM2 knockdown decreases PD-L1 expression and improves the anti-tumor efficacy of PD-1/PD-L1 blockade (Xia et al., 2022). Several newly found PKM2 inhibitors with high cancer repression *in vitro* and mice xenograft models, such as Shikonin and Isovitexin (Zhao et al., 2018; Chen et al., 2021a), need more clinical trials to detect the efficiency and toxicity of PKM2 inhibition in complex internal environments.

## Targeting lactate generation and transportation for immunotherapy

### Directly buffering the acidic tumor microenvironment

The reversibility of adverse effects on anti-tumor immunity mediated by TME acidification is the prerequisite for targeting lactate to improve anti-tumor immunity and optimize immunotherapy. A study validates that the attenuated functions of CTLs would be restored when cultured in the lactic acid-free medium (Fischer et al., 2007). The treatment of proton pump inhibitors (PPIs) can optimize adoptive transfer lymphocyte therapy by buffering the acidized TME to revert the effector T cell functions (Calcinotto et al., 2012). Moreover, bicarbonate monotherapy can slow cancer growth sufficiently in the mice model, and bicarbonate combined with ICB or ACT further improves therapeutic responses, suggesting lactate acidosis could induce immunotherapy resistance (Pilon-Thomas et al., 2016).

### Lactate dehydrogenase

LDH mediates the interconversion of lactate and pyruvate, composed of different combinations of LDHA and LDHB towards a tetramer (Decking et al., 2022). LDHA has a strong affinity for pyruvate, while LDHB binds lactate more (Decking et al., 2022). LDH controls the regeneration of NAD^+^, which will be consumed at the glyceraldehyde-3-phosphate dehydrogenase step of glycolysis. Thus, LDH is indispensable for glycolytic persistence. The research found that NAD precursors like nicotinamide riboside (NR) can reduce the mitochondrial disability in CD8^+^T cells and elicit more potent inhibition of cancers when combined with ICBs (Yu et al., 2020). Serine supplementation can poise the LDH-mediated NAD^+^/NADH redox unbalance in high lactate cellular environment to relieve the proliferation of T cells, based on the findings by Quinn et al. that redox homeostasis is a therapeutic target for developing immunotherapy (Quinn et al., 2020).

Notably, the MCT-mediated efflux of lactate from CD8^+^T cells is determined by the relative lactate gradient between intracellular and extracellular environments. Therefore, a high lactate level in TME will lead to high lactate inside stacking (Fischer et al., 2007). The intracellular acidification impairs T cell energy metabolism and functions (Brand et al., 2016), abrogating the cancer repression of effector T cells and NK cells by preventing the NFAT and IFNγ expression (Brand et al., 2016). LDHA inhibitor oxamate increases the TME infiltration of CD8^+^T cells, and the combination of oxamate and pembrolizumab shows a favorable response in NSCLC mouse models (Qiao et al., 2021). However, LDHA inhibition shows bi-directional effects on T cell functions. LDHA knockout would impair glucose consumption and lactate production in T cells, especially interrupts glycolytic ATP production and the FOXO1 expression in PI3K-AKT dependent manner (Peng et al., 2016; Xu et al., 2021). In the activated Th1 cells, LDHA promotes histone acetylation by maintaining a high cytoplasmic acetyl-CoA concentration; in this way, Th cells upregulate IFNγ expression and enter the differentiation course (Peng et al., 2016). Besides, genetic and pharmacological LDHA inhibition may induce unexpectedly metabolic rewiring in cancer cells, such as alternative metabolic pathways, including lipid metabolism and the FAO pathway. The compensatory LDHB upregulation-inducible lactate consumption can facilitate cancer aggressiveness (Maeda et al., 2022). Therefore, approaches are needed to handle LDH activation to inhibit cancer growth without inducing anti-tumor immunity destruction. In addition, LDHB overexpression also enhances the cytotoxic capacity of murine T cells in HCT116 tumor spheroids (Decking et al., 2022). These findings suggest the close crosstalk between lactate metabolism and immunity.

### Monocarboxylate transporter

MCT neutralization helps alleviate the acidification state of TME, creating a habitable surrounding for T cell infiltration and cytotoxicity. MCT1, 2, and 4 are the major transporters for products of the glycolysis cycle in cancers (Felmlee et al., 2020). Mechanistically, MCT1 inhibition enhances effector T cell infiltration and eases T cell exhaustion, further repressing the immunosuppressive function of Tregs by blocking lactate uptake and associated metabolic program. Especially in high glycolytic cancers, such as MYC-driven cancers, MCT1 facilitates lactate uptake and PD-1 expression in Tregs, a novel mechanistic view of anti-PD-1 therapy resistance (Kumagai et al., 2022). MCT1 inhibitor AZD3965 mounted on a nano drug combined with anti-PD-1 therapy can provide additional tumor suppression effects (Huang et al., 2021; Watson et al., 2021). MCT4 silencing also improves immunotherapy response in hepatocellular carcinoma (HCC) (Fang et al., 2022). Of note, MCT1 inhibitors can restrain lactate excretion from effector T cells concomitantly (Fischer et al., 2007). So similar to LDH inhibition, it is essential to determine the right MCT dose to enhance immunotherapy without disturbing prime immune surveillance.

### Other promising therapeutic targets

Preclinical studies demonstrated that the metabolic switch from glycolysis toward oxidative metabolism could enhance effector T cell anti-tumor performance. Ning et al. found that carbonic anhydrase Ⅻ (CA12) is upregulated in tumor-infiltrating monocytes and macrophages upon the glycolytic switch induced by HIF-1α. CA12 promoted macrophage survival and HCC metastasis, and CA12 inhibitors synthetically repress cancer growth and metastasis by combining with ICBs in Hepa1-6 mouse models (Ning et al., 2022).

Glucose shunted to other metabolic pathways is also helpful for glycolysis constraint. The Glucose-6-phosphate dehydrogenase (G6PD) is the rate-limiting enzyme of PPP, whose high expression will partition glucose flux from glycolysis. Lu et al. found that G6PD activator AG1 could increase H3K9 acetylation at the Gzmb locus through upregulating acetyl-CoA to enhance Gzmb expression and TILs cancer-lytic ability (Lu et al., 2022).

Glutamine metabolism is closely associated with glycolysis by supplying substrates for TCA cycle (Madden and Rathmell, 2021). Glutamine converts into α-ketoglutarate which drives the TCA cycle and synthesizes intermediates for other metabolic tracers and anabolic growth. In MYC-driven cancers, glutamine deletion impairs the TCA cycle and inhibits cancer cell viability in an energetic-demand mechanism (Edwards-Hicks et al., 2022). Instead, in T cells, the inhibition of glutamine metabolism focuses T cell adaption to glycolytic strategy and promotes their proliferation and effector differentiation (Leone et al., 2019). In addition, glutamine antagonism inhibits both glucose and glutamine metabolism in cancers, along with increasing the nutrient contents of TME, whereas CD8^+^T cells synchronously increase acetate metabolism to fuel TCA-coupled OXPHOS and achieve more prolonged survival upon glutamine inhibition (Leone et al., 2019). This divergence in metabolic plasticity to glutamine antagonism demonstrates the cancer-targeting ability of glutamine inhibition in TME.

Besides the metabolic crosstalk between glycolysis and glutamine, subsets of TILs that can not uptake glucose efficiently have high ROS levels in hyperpolarized mitochondria, and in this case, pyruvate supplementation can ignore glycolysis defects and eliminate ROS to partially reinvigorate T cells (Siska et al., 2017). Collectively, the basic metabolic interplay underpinning novel “immunometabolism” strategy development needs more elucidation to achieve better efficacy in clinical practice.

## Discussion

Glycolytic activation is a key link process of metabolism reprogramming in cancer and immune cells, tightly associated with the efficiency of anti-tumor immunity, and affects the performance of immunotherapies. We explain at length the glycolytic barriers within checkpoint inhibition and adoptive cell therapies, and emphatically summarize the critical glycolytic targets helpful for enhancing immunotherapy. However, only the tip of the iceberg has been unraveled regarding the mechanisms related to cellular metabolism in optimizing cancer immunotherapy, and large-scale studies are warranted for a deeper understanding of the crosstalk between metabolic pathways with cancer immunity.

Given the complication and systematicness of cellular metabolism regulation, it is reasonable to consider the holistic influence induced by genetic or pharmacological interference of metabolic-related molecules in future studies. Especially, due to the metabolic plasticity of cancer cells and immune cells, more attention is warranted to focus on the secondary effects of metabolic therapy, including compensatory activation of metabolic pathways and the TME reshaping. In addition, the metabolic interaction between cells in the TME may play a crucial role in cancer behavior and immunity. For example, the enhanced methionine uptake promoted by SLC43A2 upregulation in cancer cells significantly causes T cell dysfunction (Bian et al., 2020), the nutrient support of cancer-associated fibroblasts promotes cancer progression (Mostafavi et al., 2022), and the metabolic symbiosis between glycolytic cancer cells and oxidative cancer cells (Wang et al., 2021). These findings stress that metabolic cooperation and antagonism happening in TME is promising for novel target development.

In conclusion, we give a full-scale review of glycolysis in cancer immunity. We hope our work can offer novel insights into cancer metabolism and contribute to the development of novel therapeutic strategies in the era of immunotherapy.
